# Small-Area Lung Cancer Incidence and Mortality: Cross-Sectional Population-Based Study Using Hospital Discharge and Death Registration Data

**DOI:** 10.2196/74062

**Published:** 2025-09-29

**Authors:** Yu He, Xinxin Xia, Qing Wang, Yaoyun Zhang, Ying Meng, Xiaokang Ji, Qingbo Zhao, Yongchao Wang, Yifu Zhao, Fuzhong Xue, Jin Xu

**Affiliations:** 1 Department of Journal Management, Chinese Preventive Medicine Association, Beijing, China; 2 China Center for Health Development Studies, Peking University, 38 Xueyuan Road, Haidian District, Beijing, 100191, China; 3 School of Public Health, Shandong University, Jinan, China; 4 School of Public Health, Peking University, Beijing, China

**Keywords:** cancer incidence, cancer mortality, small-area analysis, administrative health data, epidemiology

## Abstract

**Background:**

Despite rapid development, cancer registries in low- and middle-income countries, such as China, have the persistent problems with up to 6-year delay and a lack of reported details about small areas.

**Objective:**

This study aimed to develop an approach to provide more up-to-date localized cancer surveillance using linked administrative data. We used lung cancer as an example.

**Methods:**

Based on data of hospitalization record front pages (HRFPs) between 2013 and 2022 from all the secondary and tertiary hospitals in Shandong Province, China, we identified incident cases of lung cancer in 2022 with 2013‐2021 being the washout period. Deaths from lung cancer were ascertained for 2022 using linked HRFPs and death registration data. We estimated age-standardized incidence and mortality rates (ASIR and ASMR) of lung cancer in 2022 using Segi world standard population, age-specific incidence and mortality rates by sex, and county-level ASIR and ASMR to illustrate regional disparity. We grouped the counties by municipalities and calculated the Theil indices for within-municipality inequality and between-municipality inequality.

**Results:**

The HRFPs captured 79,672 incident cases of lung cancer in Shandong in 2022 (45,527 males, 34,145 females). The ASIR of lung cancer in Shandong was 42.46 per 100,000 in both sexes (49.19/100,000 in males vs 36.67/100,000 in females). A total of 40,626 lung cancer-specific deaths were ascertained (28,185 men and 12,441 women). The ASMR was 19.76/100,000 in both sexes, 26.29/100,000 and 11.38/100,000 in males and females, respectively. The IQR of county-level ASIR and ASMR were 17.13/100,000 and 10.41/100,000, respectively. The inequality was primarily due to within-municipality disparities, with within-municipality Theil T indices higher than between-municipality Theil T indices (0.0572 vs 0.0033 for ASIR, 0.0824 vs 0.0011 for ASMR).

**Conclusions:**

The cancer surveillance approach based on linked administrative data could provide up-to-date small-area estimates of cancer burden, when cancer registry data are not yet reported and for areas not covered by cancer registries. It could reveal disparity of cancer epidemiology, which provides leads for further investigation into the underlying causes and potential solutions for equity improvement.

## Introduction

With an estimated 20 million new cancer cases and 9.7 million cancer deaths in 2022 worldwide, cancer ranks as the third contributor to the global burden of disease [1]. The role of cancer registries as a key source of information for surveillance of cancer burden to reveal the need for global cancer care has been well recognized [2]. At the national and subnational level, timely and locally accurate information about the incidence and mortality of cancer enables the identification of high-risk areas, monitoring of the effectiveness of health interventions, and development of the targeted solutions to address a broad range of determinants of health, potentially diverse across areas [3,4]. Despite the rapid development of cancer registries in low- and middle-income countries like China, challenges remain regarding delayed reporting [5,6], issues with data accessibility [7-9], and population coverage [10].

With the growing availability of high-quality data based on hospitalization records and other forms of administrative data [11], it is both important and feasible to attempt alternative or complementary approaches to cancer surveillance beyond cancer registries. Indeed, medical insurance claims data have been demonstrated as a reliable source for cancer surveillance [12,13]. Similarly, researchers have also proposed electronic health records, hospital record data in particular, for cancer surveillance as a supplement to or in the absence of a cancer registry [14,15]. Studies from Canada, Spain, and Australia examined the performance of incident hospitalization record in estimating cancer incidence against cancer registries and found high concordance between the two, especially for particular cancer types such as lung cancer and colorectal cancer [16-18]. With the increasing availability of administrative health care data, their use may offer a more detailed and timely surveillance of cancer epidemiology that can inform more effective resource allocation and policy making.

We selected lung cancer as the example in this study due to the heavy burden of this disease. Among all the types of cancer, it has long been the leading cause of both new cancer cases (age-standardized incidence rate [ASIR] in 2022: 40.78/10,000) and cancer deaths (age-standardized mortality rate [ASMR] in 2022: 26.66/10,000) in China [6]. In Shandong Province in particular, lung cancer has been the top cause of cancer death since 2004 [19].

In this study, we sought to develop a timely approach to estimating small-area lung cancer incidence and mortality rates for one of the most populous provinces in China and its counties by linking population-based hospital discharge records and death registration. A small area, depending on the purpose and data availability of the study, may be a neighborhood, a census tract, or a county [20]. In this study, the geographic scale of a small area refers to a county. We also mapped the county-level lung cancer incidence and mortality rates to evaluate the degree of intercounty disparity of lung cancer incidence and mortality.

## Methods

### Study Setting

Shandong Province is located on the eastern coast of northern China, covering an area of 157,100 km^2^. It had a population of 101.6 million people, or about 7.2% of China’s total population in 2022 [21]. The province’s gross domestic product per capita in the same year was 86,143 Chinese yuan (or US$12,800), equivalent to the level of an upper-middle–income economy by the World Bank categorization [22-24]. Qualified cancer registry data in Shandong covered approximately 33.26 million registered residents, accounting for about 33.24% of the province’s total population [25]. Within the province, there are 17 municipalities which in turn consist of 137 counties (including districts and county-level cities). The eastern coastal areas in Shandong are economically more developed, especially Qingdao Municipality and its neighboring municipalities (Weihai, Yantai, Weifang, and Rizhao), compared with other areas in Shandong [26].

### Data Sources

This study used the hospitalization record front pages (HRFPs) data from all secondary and tertiary hospitals in Shandong and the death registry covering the whole population in the province. The HRFP covered a wide range of information, including patient demographics, residence, health-related, and hospitalization-related information, among others. Both datasets were stored in the Cheeloo Lifespan Electronic Health Research Data-library (Cheeloo LEAD), which is an electronic health data platform linked via encrypted individual identity numbers [27] . We extracted the following data from HRFP from the Cheeloo LEAD between January 1, 2013, and December 31, 2022: sex, year of birth, date of admission, county of residence, and diagnosis upon admission recorded in *International Classification of Diseases, Tenth Revision (ICD-10)* code [28]. We further extracted records for people who died between January 1, 2022, and December 31, 2022, from the death registers, covering sex, year of birth, county of residence, and cause of death (also based on *ICD-10* code). These data were complemented by county-level and provincial population data from the latest census reported by Shandong Provincial Bureau of Statistics [29].

This article followed the Strengthening the Reporting of Observational Studies in Epidemiology (STROBE) reporting guideline [30] (see Checklist 1).

### Identification of Incident Lung Cancer Cases and Deaths

We identified lung cancer–related hospitalization using *ICD-10* codes from C34.0 to C34.9. To avoid classifying prevalent cases as incident cases, we excluded cancer patients with hospitalization records due to lung cancer in previous years (ie, 2013‐2021). In other words, we adopted a washout time window from 1 year up to 9 years [2]. While a longer washout period appeared to generate more precise results, the benefit of an extra washout year beyond 1 year appeared nonsubstantial (see Figure S1 in Multimedia Appendix 1). For patients with records of multiple hospitalizations for lung cancer, an incident lung cancer case was defined based on the first definite lung cancer–related hospitalization.

We used the corresponding date of admission as the date of diagnosis [31]. As the number of cases with missing information for each of the variables listed in the previous section was below 10, we removed the cases with missing values (a total of 18 cases were excluded).

We ascertained lung cancer–specific death cases based on lung cancer–related records in the death registers from January 1, 2022, to December 31, 2022, with the corresponding registration date as the date of death. *ICD-10* codes were used to determine the primary cause of death. We excluded 1 death case with missing values.

**Figure 1.**
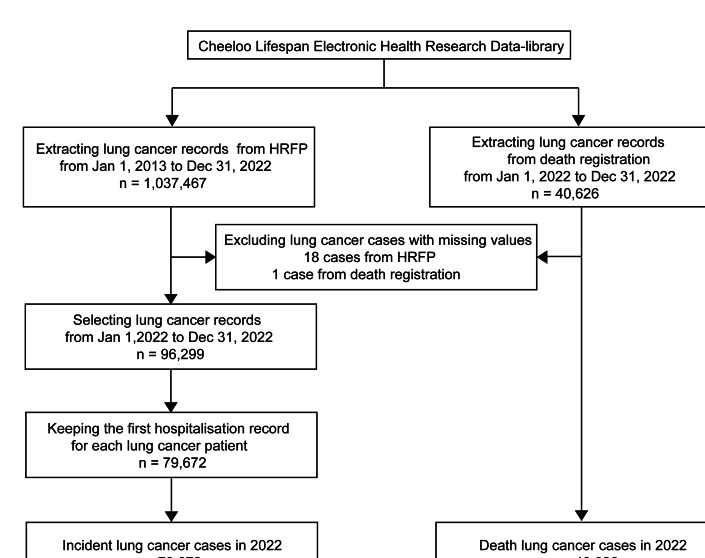
Identification of incident and death lung cancer cases in Shandong, China, 2022. Hospitalization record front pages were identified as lung cancer with diagnoses in *International Classification of Diseases, Tenth Revision* codes from *C34.0* to *C34.9*. Records from death registration were identified as lung cancer-related death with cause of death in *ICD-10* codes from *C34.*0 to *C34.9*.

The procedure of identifying incident lung cancer cases and deaths from lung cancer in Shandong in 2022 is shown in Figure 1.

### Statistical Analysis

We evaluated crude, age-specific, and ASIR of lung cancer in Shandong in 2022 by gender with the corresponding resident population as the denominator (structure of the resident population in Shandong in 2022 by gender is shown in Figure S2 in Multimedia Appendix 1), using the formulas below.


$$\text{Crude incidence rate}=\frac{\text{New lung cancer cases in the year of interest}}{\text{Total population in the same year}}\times 100,000$$

$$\text{Age-specific incidence rate}=\frac{\text{New cases of a disease in a given age group}}{\text{Total population in the same age group}}\times 100,000$$

$$\text{Age-standardised incidence rate}=\frac{\sum \left(\text{Age-specific incidence rate}\times \text{Standard population in corresponding age group}\right)}{\sum \text{Standard population}}\times 100,000$$

Age-standardization was based on Segi world standard population. Crude, age-specific, and ASMR were calculated following a similar method.

Lung cancer incidence and mortality were also calculated at the municipal and county levels. We calculated the difference between the third quartile (Q3) and the first quartile (Q1) as the IQR to quantify geographical disparity in age-standardized incidence and mortality rates of lung cancer at the county and municipality levels. In addition, we used the Theil index to estimate the overall inequality in these rates [32,33], because it could be disaggregated. We grouped the counties based on municipalities where they were located and calculated the Theil indices (Theil T and Theil L) for these groupings, estimating both within-municipality inequality (disparities between counties within each municipality) and between-municipality inequality (disparities between municipalities). A higher Theil index indicates greater inequality [34]. Details about calculating Theil T and Theil L can be found in xMethods Part S2 in Multimedia Appendix 1.

We also evaluated the internal validity of this cancer surveillance method by examining the stability of incidence rates over the years from 2019 to 2022, referring to a criterion on data quality by the International Agency for Research on Cancer [35]. The same formulas for estimating the ASIR and ASMR of lung cancer in the year 2022 were adopted for the years 2019 to 2022. Based on the previously reported lung cancer incidence time trends [36], we assumed a threshold of an annual growth rate of 10% as indicating the stability of the data over time. The external validity was assessed by comparing the estimates derived by the method in this study with the cancer statistics reported by the national cancer registry in China for the same time period.

All statistical analyses in this study were performed using R software (version 3.4.1; R Foundation for Statistical Computing), with maps also generated using R software.

### Ethical Considerations

This study received ethical approval from the Ethics Committee for Public Health of Shandong University (registration number LL20241105). As the study used preexisting, deidentified secondary data, the Ethics Committee waived the requirement for informed consent. Before analysis, all personally identifiable information was removed and replaced with anonymous identifiers. The privacy and confidentiality of all research subjects were strictly maintained. No compensation was provided to the participants.

## Results

### Number of Incident Cases and Incidence Rate of Lung Cancer at the Provincial Level

**Figure 2.**
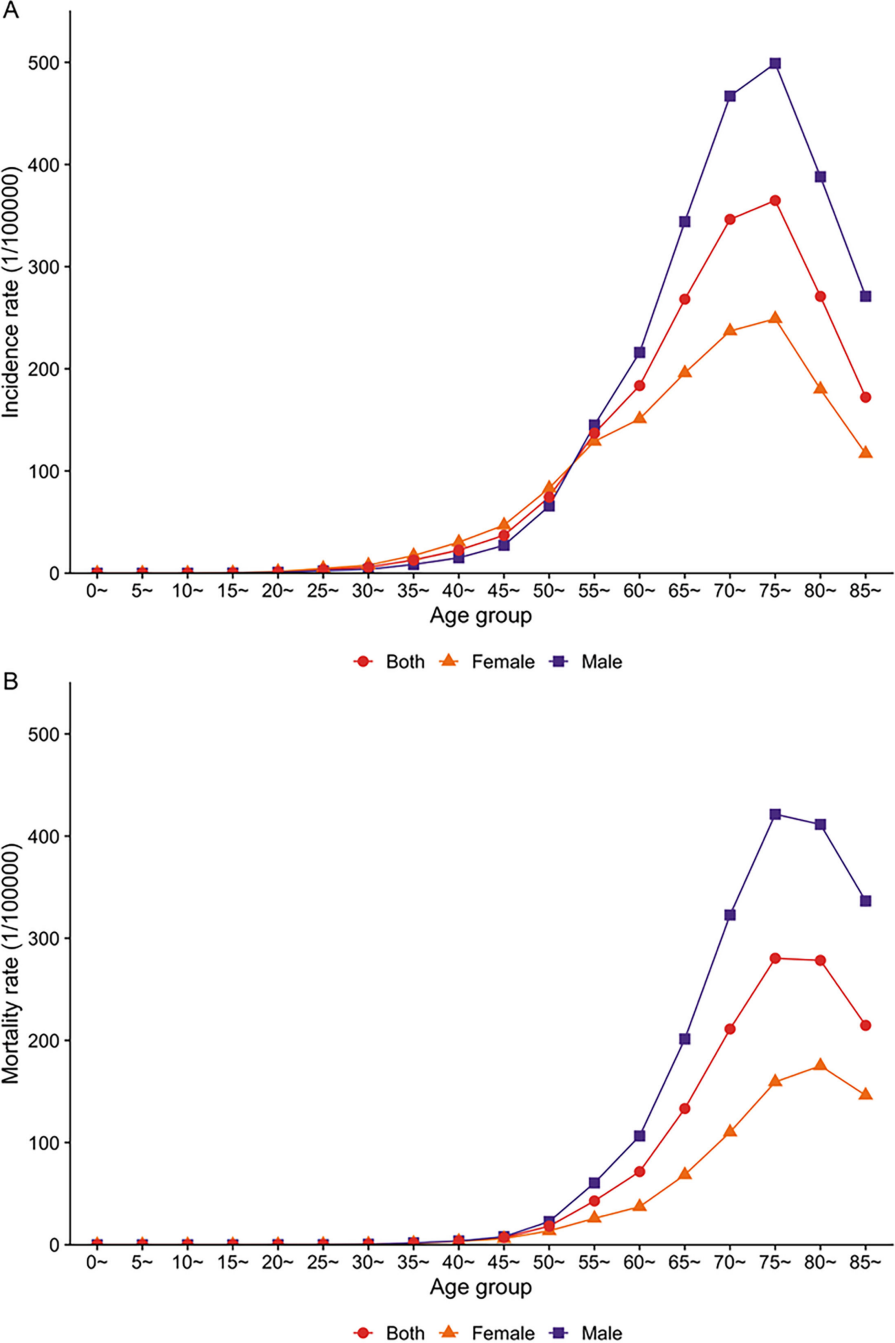
Age- and sex-specific incidence and mortality of lung cancer in Shandong, China, 2022, estimated using hospital discharge and death registration data. (A) Age- and sex-specific incidence rate of lung cancer. (B) Age- and sex-specific mortality rate of lung cancer.

In 2022, there were an estimated 79,672 incident cases of lung cancer in Shandong, including 45,527 males and 34,145 females. The estimated crude incidence of lung cancer in 2022 was 78.40 per 100,000 people. Crude lung cancer incidence rate in males (88.35 per 100,000) was higher than in females (68.15 per 100,000). The ASIR of lung cancer using Segi population was 42.46 per 100,000. The ASIR was 49.19 per 100,000 in males and 36.67 per 100,000 in females, resulting in a male-to-female ratio of 1.34 (Figure 2A).

The incidence of lung cancer rose rapidly after age 50 years, peaked around 75, and gradually dropped in people older than 75 in both men and women. Only in age groups between 35 and 50 was the incidence of lung cancer higher in females than in males.

From 2019 to 2022, the annual growth rate in the lung cancer ASIR in Shandong was within 10%, indicating the reliability of the lung cancer incidence estimates over time (Table S1 in Multimedia Appendix 1).

### Number of Deaths and Mortality Rate From Lung Cancer at the Provincial Level

A total of 40,626 lung cancer-specific deaths were ascertained in Shandong in 2022, among which 28,185 were men and 12,441 were women. The estimated crude mortality of lung cancer in 2022 was 39.98 per 100,000 people (54.69 per 100,000 in males, and 24.83 per 100,000 in females). The ASMR of lung cancer using the Segi population was 19.76 per 100,000, which was higher in males than in females (26.29 per 100,000 vs 11.38 per 100,000; male-to-female ratio: 2.56).

Age-specific mortality from lung cancer started increasing after age 50 years and peaked in the age group 75‐80 years in both sexes (Figure 2B). Except for age groups younger than 45 years, in which both men and women experienced a mortality close to zero, the mortality was consistently higher in males compared with females.

### Geographical Disparity of Incidence and Mortality at the County Level

**Figure 3.**
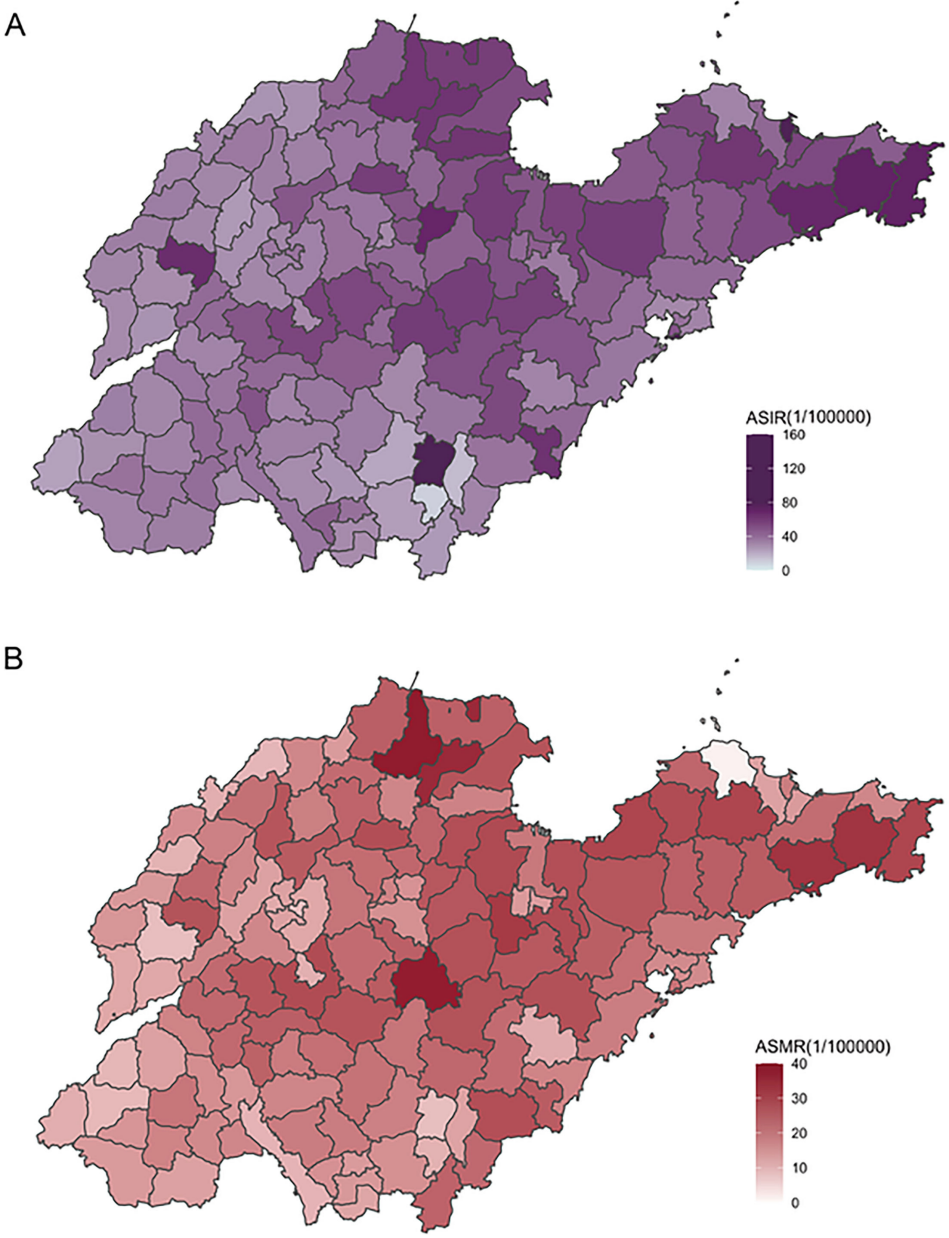
Geographical distribution of (**A**) Age-standardized incidence and (**B**) Mortality rate of lung cancer among county-level divisions in Shandong, China, 2022. ASIR; age-standardized incidence rate; ASMR: age-standardized mortality rate.

The IQR was substantial for county-level ASIR at 17.13 per 100,000 people (Table 1). The Theil index for the ASIR and ASMR of lung cancer was 0.0605 and 0.0835 for Theil T, and 0.0586 and 0.0787 for Theil L, respectively (Table 2). The overall inequality was primarily due to within-municipality disparities, with the within-municipality Theil index accounting for 0.0572 for ASIR and 0.0824 for ASMR in Theil T, and 0.0261 for ASIR and 0.0295 for ASMR in Theil L. The minimal county-level ASIR was observed in Luozhuang of Linyi Municipality (southern Shandong) at 9.50 per 100,000 people, while the maximum was observed in Shinan of Qingdao Municipality (eastern Shandong) at 158.16 per 100,000 people (Figure 3A; see Figure S3A, S4A, and S5A in Multimedia Appendix 1 for maps for crude county-level incidence rate, crude municipal-level incidence rate, and municipal-level ASIR, respectively. Meanwhile, the IQR of municipal-level ASMR was smaller (10.41 per 100,000 people). The minimal county-level ASMR was observed in Penglai of Yantai Municipality (northeastern Shandong) at 1.41 per 100,000 people, while the maximum was observed in Zhanhua of Binzhou Municipality (northern Shandong) at 38.62 per 100,000 (Figure 3B; see Figure S3B, S4B, and S5B in Multimedia Appendix 1 for maps for crude county-level mortality rate, municipal-level mortality rate, and municipal-level ASMR, respectively).

**Table 1.** Quartiles and IQRs for age-standardized incidence and mortality rates of lung cancer (per 100,000 people) in Shandong, China, 2022.

Level	ASIR^a^, median (IQR)	ASMR^b^, median (IQR)	ASIR, (minimum–maximum)	ASMR, (minimum–maximum)
County-level	38.70 (31.91–49.04)	19.63 (14.71–25.12)	9.50–158.16	1.41–38.62
Municipal-level	41.40 (35.93–49.36)	20.01 (16.86–23.28)	31.01–59.80	13.53–26.17

a ASIR: age-standardized incidence rate.

b ASMR: age-standardized mortality rate.

**Table 2.** Decomposition of intercounty inequality of age-standardized incidence and mortality rates of lung cancer (per 100,000 people) in Shandong, China, 2022.

Component	ASIR^a^	ASMR^b^
Overall intercounty inequality		
Theil T	0.0605	0.0835
Theil L	0.0586	0.0787
Between-municipality inequality		
Theil T	0.0033	0.0011
Theil L	0.0325	0.0492
Within-municipality inequality		
Theil T	0.0572	0.0824
Theil L	0.0261	0.0295

a ASIR: age-standardized incidence rate.

b ASMR: age-standardized mortality rate.

## Discussion

### Principal Findings

This study established a novel approach to cancer surveillance by linking data from hospital discharge records and death registries. Using this approach, we estimated that the age-standardized lung cancer incidence and mortality rates were 42.46 per 100,000 and 19.76 per 100,000, respectively, in Shandong, China, in 2022. The IQR of county-level ASIR was 17.13 per 100,000, while the IQR of county-level ASMR was 10.41 per 100,000. The analyses based on Theil indices suggested that the regional disparity of ASIR and ASMR of lung cancer was primarily due to inequality between counties within each municipality. Hotspots of high ASIR and ASMR counties were also identified. This study demonstrates the potential of using routinely collected administrative hospital records in estimating local cancer incidence and death, offering a reliable approach for up-to-date and small-area cancer surveillance in the future.

For internal validation, we assessed the ASIR and ASMR from 2019 to 2022 and found the rates stable during the period (ASIR: average growth rate 2.242% in males, 6.114% in females; ASMR: average growth rate 2.944% in males, 3.289% in females; see Table S1 in Multimedia Appendix 1). Regarding external validation, we found the lung cancer ASIR and ASMR in Shandong in 2022, we estimated (42.46 per 100,000 and 19.76 per 100,000), comparable to those from the national prediction for the same year by the National Cancer Center based on cancer registry data 2010‐2018 (40.78 per 100,000 and 26.66 per 100,000, respectively) [6]. Similar to the national level estimates, the lung cancer ASIR and ASMR we found were higher in males than in females, with a male-to-female ASIR ratio of 1.34 (1.71 in the national cancer report) and ASMR ratio of 2.56 (2.69 in the national cancer report) [6]. As mentioned earlier, the estimated annual growth rates from 2019 to 2022 for ASIR using this approach were 10% and thus considered stable [37]. Therefore, our findings are in line with previous studies that demonstrated the comparability of hospital discharge data and cancer registry data [16-18].

The analysis in this study revealed within-municipality disparities in lung cancer estimates. High ASIRs and AMSRs were found in counties to the north and east of Shandong Province (see Figure 3), where the economic development level is relatively higher compared with other parts of the province. This association with regional socio-economic status is similar to that in a previous study on global lung cancer burden, which reported positive correlations between ASIR and ASMR and human development index [38]. A possible explanation might be the regional difference in tobacco smoking prevalence, as a survey in 2016‐2017 in Shandong found a higher prevalence of smoking in the eastern part compared with the middle area [39]. Air quality and access to diagnostic services might also play a role [40]. Yet there is a lack of evidence on the correlation of those factors with lung cancer incidence and mortality on the geographic scale of small areas in China, which may be explored in the future, for example, in Shandong via linking the data in this study with data sources on the distribution of potential factors such as tobacco smoking exposure, air pollution, etc.

Our approach to rapid and local cancer surveillance by linking two administrative datasets has some advantages that warrant its consideration as a complement to cancer registries. First, it allows the rapid estimates of cancer burden. In this study, we used the records up to 2022 to calculate the incidence and mortality in 2022. It achieved much higher timeliness compared with the 6-year delay of cancer registry data [41]. Compared with medical claims data [2], for which a 6-month delay was expected owing to the lag between discharge and reimbursement, our approach using hospital discharge records could be captured in real-time without delay. Indeed, with more up-to-date data, even more timely estimation can be achieved. Second, the hospital discharge records were generated and reviewed by health care professionals during hospitalization and heavily scrutinized by the health administration, which ensured high data quality as demonstrated in previous studies [42-44]. Third, linked records from all hospitals in an area of interest achieved an almost universal coverage of local residents, while the cancer registries in Shandong only covered 33.24% of its population [31]. It is true that if a patient visited hospitals outside Shandong Province, the corresponding record would not be captured in this study. Yet, due to geographical access and higher reimbursement, patients with cancer were generally diagnosed at health care facilities not far from their residences. Therefore, the impact of multiple hospital visits on the identification of incident cancer cases was considered not leading to substantive underestimation of the results in this study. In areas where the cancer registry sites are yet to be established, the approach using routinely collected hospital discharge records may provide reasonably comprehensive cancer statistics. This also offers a potentially powerful solution for generating high-resolution cancer statistics using existing data in China and other low- and middle-income countries.

While administrative data harbor great potential in disease surveillance, among other health-related topics, there are multiple barriers to use administrative data of sufficient volume for research [45]. First of all, the health-related records may not be digitized and structured into a dataset available for data analysis and linkage, especially in low-resource settings. In addition, data linkage algorithms, data accuracy, and coding standardization make synthesizing data across different institutions/regions a challenging project [46,47].

### Limitations

This study has some limitations that warrant consideration when generalizing the findings. First, people who only sought cancer care outside Shandong would be missed. This issue would be magnified in an area lacking cancer-specialty resources. However, given that Shandong is a large middle-income province in China with sufficient cancer treatment capacity, it is highly unlikely that a substantial proportion of residents with cancer would rely purely on inpatient care outside the province. Second, outpatient cancer care was not included in this study. However, for an illness as severe as cancer, diagnostic tests, investigations, and confirmed diagnosis can rarely be completely provided in ambulatory settings, and the Chinese Lung Cancer Clinical Guidelines recommend hospitalization for surgery in almost all cases [43]. Hence, this limitation is unlikely to lead to a serious underestimation of the cancer incidence.

### Implications

Our study has some potential implications. First, we have demonstrated the feasibility of producing timely and small-area cancer incidence and mortality rates for a large population in a low- and middle-income setting. Considering the economic disparity within the province, our approach to cancer surveillance would be potentially suitable for a wide range of countries and areas [48]. Second, the increasing availability and standardization of administrative data means that administrative data should be increasingly made use of in generating more timely and spatially precise estimation of cancer incidence and mortality [49-51]. Third, the obvious disparity within the province (particularly within municipalities) suggests the importance of local level surveillance, policy making, and resource allocation that can reveal and address such geographical inequality. Fourth, linking claims data with hospital records and death registry where possible in the future could further improve the accuracy of estimation. It is also recommended that a comparison between the estimation of cancer incidence and mortality using our approach and cancer registries be conducted to further validate this approach based on linked administrative health data.

### Conclusions

This study developed and validated a timely and spatially precise cancer surveillance method for a province of 100 million population in China using administrative data. The ASIR of lung cancer was 42.46 per 100,000, with a between-county IQR of 17.13 per 100,000. Meanwhile, the ASMR of lung cancer was 19.76 per 100,000, with a between-county IQR of 10.41 per 100,000. Besides providing new insights for cancer prevention and control in China, our study demonstrates the power of a novel approach linking hospital discharge records with death registries in enabling timely and geographically sensitive cancer surveillance. The fact that such data were routinely generated in this low- and middle-income setting with large population implies wide applicability as a complement to cancer registries.

## Supplementary material

10.2196/74062 Multimedia Appendix 1 Supplementary materials.

10.2196/74062 Checklist 1 STROBE checklist.

## Abbreviations

ASIR: age-standardized incidence rate
ASMR: age-standardized mortality rate
Cheeloo LEAD: Cheeloo Lifespan Electronic Health Research Data-library
HRFP: hospitalization record front page
*ICD-10*: *International Classification of Diseases, Tenth Revision*
STROBE: Strengthening the Reporting of Observational Studies in Epidemiology
