# Supplementary material for: Small-Area Lung Cancer Incidence and Mortality: Cross-Sectional Population-Based Study Using Hospital Discharge and Death Registration Data
Source: JMIR Public Health Surveill. 2025 Sep 29;11:e74062. doi: 10.2196/74062 (PMC12478967; doi:10.2196/74062)
Supplement: Multimedia Appendix 1 [file publichealth-v11-e74062-s001.docx]

# Methods to calculate of crude, age-specific, and age-standardized incidence and mortality rates.

1. Calculation of Crude Incidence/Mortality Rate (per 100,000 population)

$$Crude incidence rate=\frac{New lung cancer cases in the year of interest}{Total population in the same year} \times100,000$$

$$Crude mortality rate=\frac{Lung cancer deaths in the year of interest}{Total population in the same year} \times100,000$$

2. Calculation of Age-specific Incidence and Mortality Rate (per 100,000 population)

$$Age-specific incidence rate=\frac{New lung cancer cases in a given age group}{Total population in the same age group} \times100,000$$

$$Age-specific mortality rate=\frac{Lung cancer deaths in a given age group}{Total population in the same age group} \times100,000$$

3. Calculation of Age-standardized Incidence and Mortality Rates (per 100,000 population)

$$Age-standardised incidence rate =\frac{\sum Age-specific indicence rate \times Standard population in corresponding age group}{\sum Standard population} \times100,000$$

$$Age-standardised mortality rate =\frac{\sum Age-specific mortality rate \times Standard population in corresponding age group}{\sum Standard population} \times100,000$$

# Methods to calculate Theil T and Theil L indices for measuring inequalities in lung cancer incidence and mortality rate.

1. Calculation of Theil T index

$$T=\frac{1}{N}\sum_{i=1}^{N} \frac{x_{i}}{\bar{x}}\ln\left( \frac{x_{i}}{\bar{x}} \right)$$

Theil T index can be decomposed into the inequalities between groups ($T_{B}$) and inequalities within groups ($T_{W}$).

$$T=T_{B}+T_{W}$$

$$T_{B}=\sum_{k=1}^{K} y_{k}\ln\left( \frac{\bar{x}_{k}}{\bar{x}} \right)$$

$$T_{W}=\sum_{k=1}^{K} y_{k}\sum_{n=1}^{n_{k}} y_{ik}\ln\left( \frac{x_{ik}}{\bar{x}_{k}} \right)$$

Where $y_{k}$ is $k$th group’s incidence rate, $\bar{x}_{k}$ is the mean incidence of group $k$, $y_{ik}$ represents the incidence share of the ith county in the $k$th city, and $x_{ik}$ stands for th ith county’s incidence in group $k$.

1. Calculation of Theil L index

$$L=\frac{1}{N}\sum_{i=1}^{N} \ln\left( \frac{\bar{x}}{x_{i}} \right)$$

Theil L index can be decomposed into the inequalities between groups ($L_{B}$) and inequalities within groups ($L_{W}$).

$$L=L_{B}+L_{W}$$

$$L_{B}=\sum_{k=1}^{K} N_{k}\ln\left( \frac{\bar{x}}{x_{k}} \right)$$

$$L_{W}=\sum_{k=1}^{K} N_{k}\sum_{n=1}^{n_{k}} N_{ik}\ln\left( \frac{\bar{x}_{k}}{x_{ik}} \right)$$

Where $N_{k}$ and $N_{ik}$ are the $k$th group’s group size proportion of overall sample and $i$th county’s proportion of $k$th group, repectively.

**
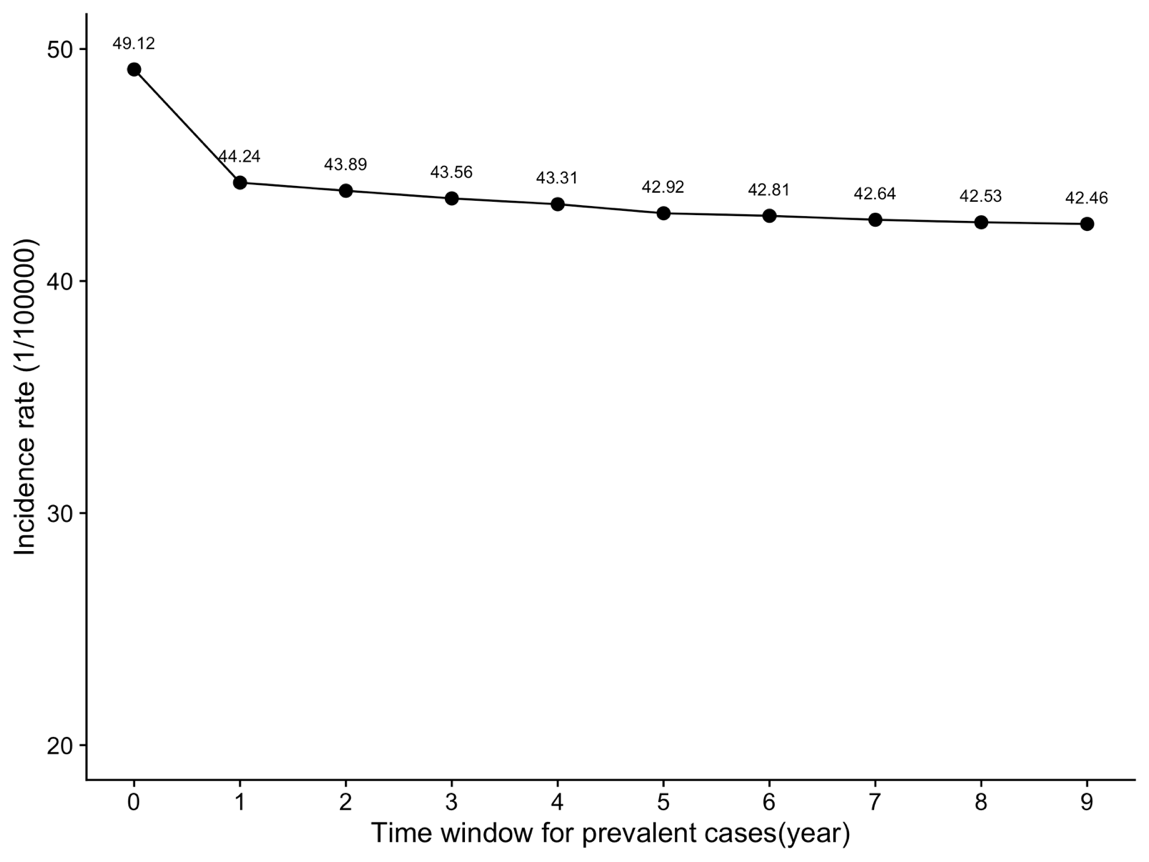
**

# Supplementary Figure S1. Estimation of age-standardized incidence of lung cancer in Shandong in 2022, using alternative washout time windows for prevalent cases.


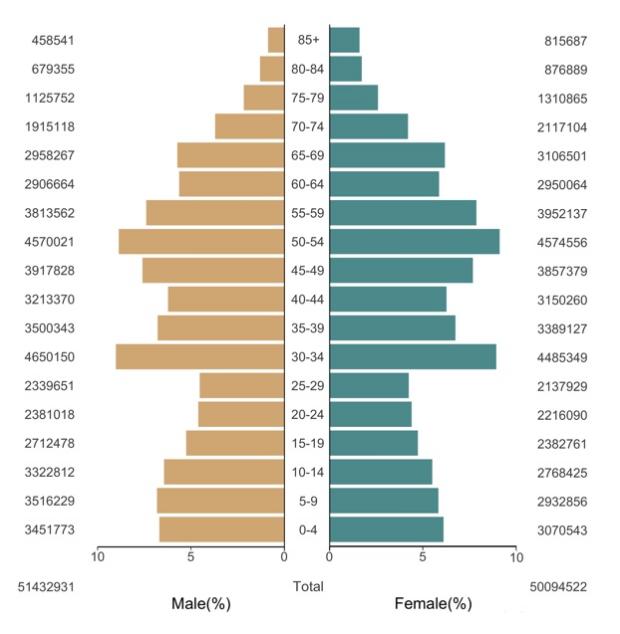


Supplementary Figure S2. Population pyramid of resident population in Shandong Province.

Source of data: the Seventh National Population Census in China, 2020


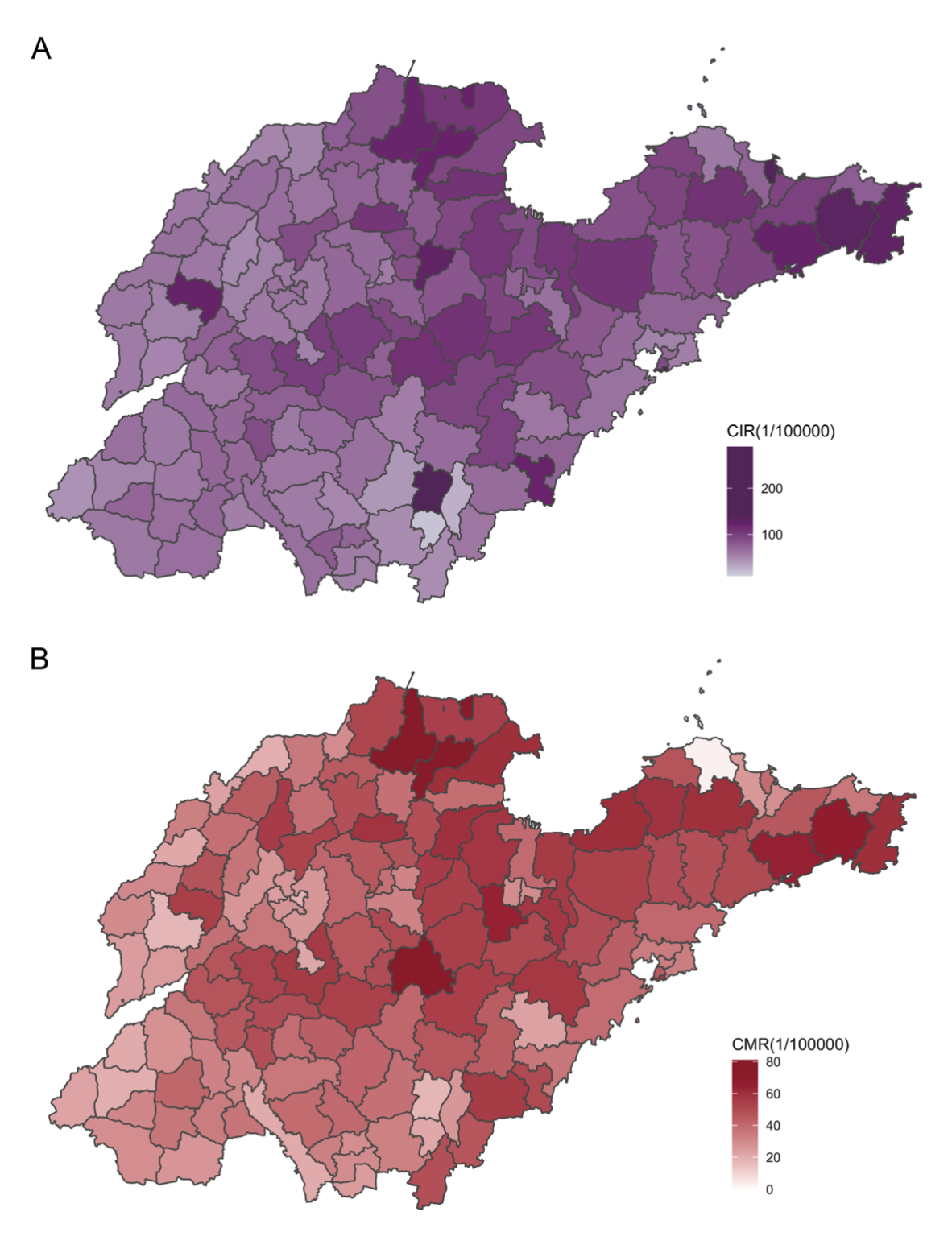


Supplementary Figure S3. Geographical distribution of (A) crude incidence and (B) mortality rate of lung cancer among county-level divisions in Shandong, 2022. CIR = crude incidence rate; CMR = crude mortality rate.


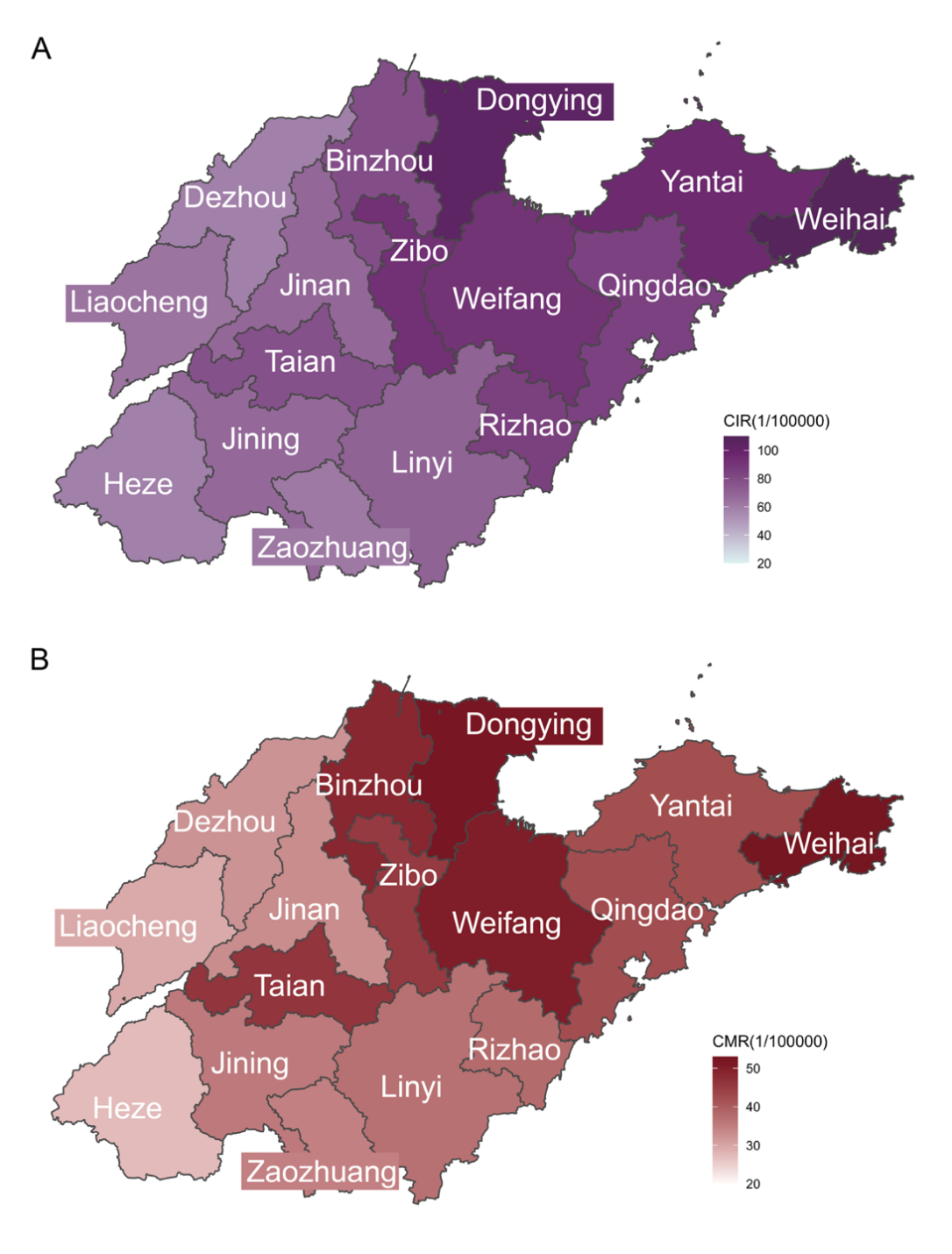


Supplementary Figure S4. Geographical distribution of (A) crude incidence and (B) mortality rate of lung cancer among municipal-level divisions in Shandong, 2022. CIR = crude incidence rate; CMR = crude mortality rate.


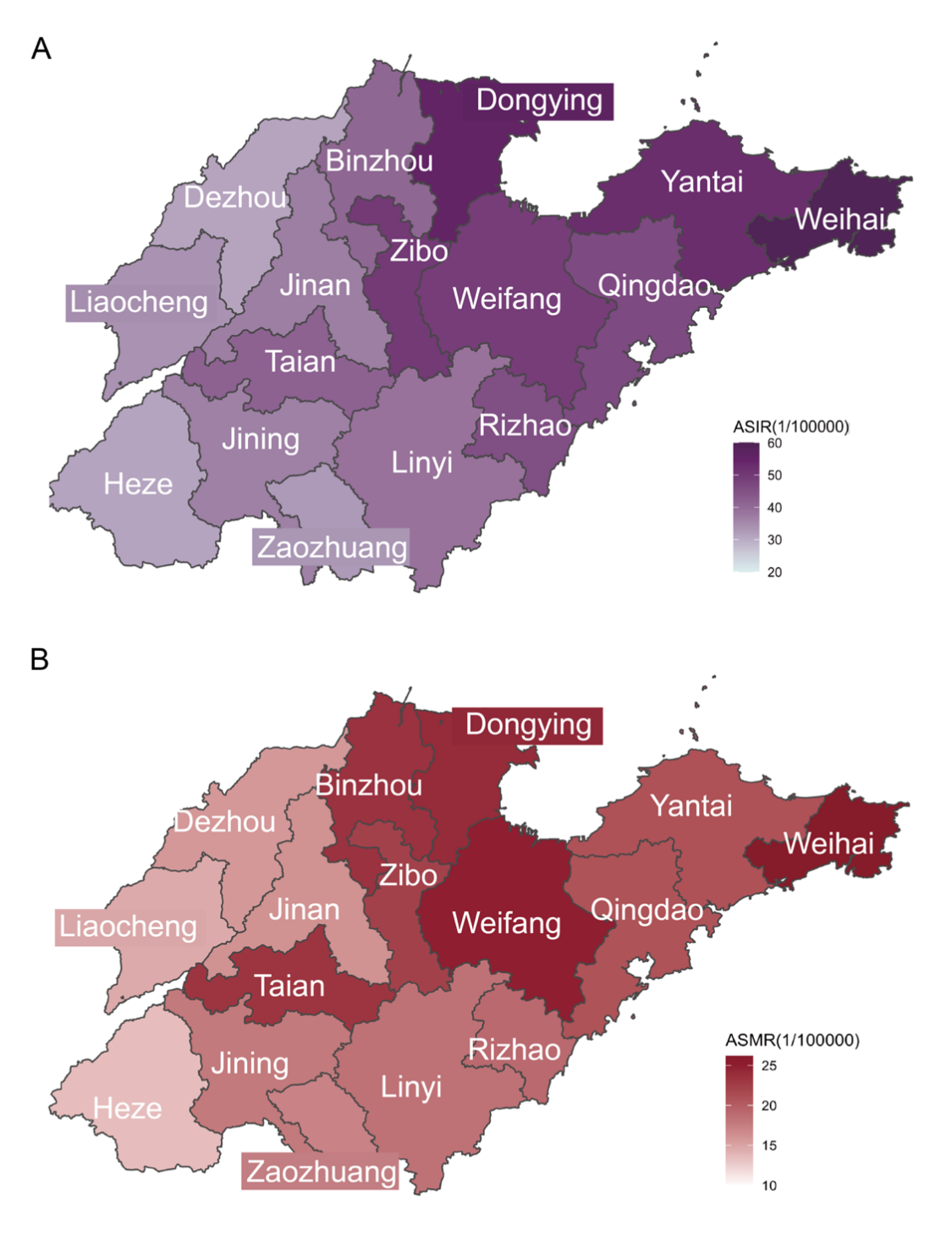


Supplementary Figure S5. Geographical distribution of (A) age-standardized incidence and (B) mortality rate of lung cancer among municipal-level divisions s in Shandong, 2022. ASIR = age-standardized incidence rate; ASMR = age-standardized mortality rate.

Supplementary Table S1. Annual change of incidence and mortality rates of lung cancer by gender in Shandong, 2019 to 2022.

| Year | ASIR (1/100,000) | | ASMR (1/100,000) | |
| --- | --- | --- | --- | --- |
|  | Male | Female | Male | Female |
| 2019 | 45.019 | 28.922 | 23.411 | 9.994 |
| 2020 | 47.314 | 32.764 | 23.092 | 9.958 |
| 2021 | 52.089 | 38.319 | 24.181 | 10.433 |
| 2022 | 49.194 | 36.670 | 26.292 | 11.375 |
| CAGR (%) | 2.242% | 6.114% | 2.944% | 3.289% |

ASIR: age-standardized incidence rate; ASMR: age-standardized mortality rate; CAGR: compound annual growth rate.
